# Carbonic anhydrase 12 mutation modulates membrane stability and volume regulation of aquaporin 5

**DOI:** 10.1080/14756366.2018.1540475

**Published:** 2018-11-19

**Authors:** Soyoung Hwang, Jung Yun Kang, Min Jae Kim, Dong Min Shin, Jeong Hee Hong

**Affiliations:** a Department of Physiology, College of Medicine, Gachon University, Incheon, Republic of Korea;; b Department of Oral Biology, BK21 PLUS Project, College of Dentistry, Yonsei University, Seoul, Republic of Korea;; c Department of Health Sciences and Technology, GAIHST, Gachon University, Incheon, Republic of Korea

**Keywords:** Carbonic anhydrase 12, aquaporin 5, volume regulation, acidosis, salivary glands

## Abstract

Patients carrying the carbonic anhydrase12 E143K mutation showed the dry mouth phenotype. The mechanism underlying the modulation of aquaporin 5 and function in the salivary glands by carbonic anhydrase12 remains unknown. In this study, we identified the mislocalised aquaporin 5 in the salivary glands carrying the E143K. The intracellular pH of E143K cells was more acidic than that of the cells carrying wild type. To evaluate the role of carbonic anhydrase12 on the volume regulation of aquaporin 5, the submandibular gland cells were subjected to hypotonic stimuli. E143K enhanced the extent of swelling of cells on hypotonicity. Aquaporin 5 modulates water influx through ion transporters to prevent osmotic imbalance. These results suggest that the carbonic anhydrase12 E143K, including acidification or inflammation, mediates volume dysregulation by the loss of aquaporin 5. Thus, carbonic anhydrase12 may determine sensible effects on the cellular osmotic regulation by modulating aquaporin 5.

## Introduction

Aquaporin 5 (AQP5) is a member of the transmembrane protein channels involved in transcellular water permeability and a dominant water channel in the epithelia of the salivary and lacrimal glands and the lung alveoli^1^^,^^2^. A study using AQP5 knockout mice showed that AQP5 plays a central role in salivary secretion^3^. Indeed, AQP5 classically prevents excessive water reabsorption and modulates cell shape and volume^4^. Recently, several new roles of AQP5, in addition to water transport, have been discussed. AQP5 is involved in migration by activating Ras and Rac signalling in cancer cells^5^ and is associated with cancer cell invasion and tumour metastasis through the NF-κB signalling pathway^6^. Additionally, hyperosmotic stress-mediated AQP5 expression mediates the expression of inflammatory cytokines and cell death through the JNK1/2 MAPK signalling pathway^7^.

The *in vitro* trafficking of AQP5 from the intracellular segments to the plasma membrane was mediated by secretory stimulation caused by factors such as acetylcholine^1^. However, *in vivo* evidence for the trafficking of AQP5 remains unknown. We focused on patients carrying the carbonic anhydrase 12 (CA12) E143K point mutation who showed dry mouth phenotype^8^. In addition, in the *ex vivo* mouse model, the volume of saliva secreted from the salivary glands of CA12 E143K (E/K)-mutated mice was dramatically reduced by using the adenovirus-mediated CA12 E/K gene transfer system^8^. Downregulated activities of the electrogenic sodium-bicarbonate co-transporter 1-B (NBCe1-B) and anion exchanger 2 (AE2) could be partially responsible for the reduced salivary volume caused by the CA12 E/K mutation. The higher electrolyte concentration caused by the CA12 E/K mutation indicated reduced water volume^8^. CA12 is a basolateral membrane-associated enzyme, which possesses the catalytic site at the extracellular surface, regulating extracellular HCO_3_^−^ concentration^9^. Typically, the membrane channels of exocrine glands such as salivary glands are localised with polarisation. AQP5 is expressed at and localised to the luminal membrane of acinar cells^10^. However, the association between CA12 mutation and reduced salivary volume remains unknown. In our previous study, we showed that the CA12 mutation mediates abnormal localisation of CA12 in the cytosol^8^. CAs generate the acidic component, CO_2_, as well as the pH buffer ion HCO_3_^−^^9^. The cytosolic expression of CA12 E/K mutation can be expected to be modulated by the intracellular pH (pH_i_). CA12 mutation-induced modulation of the water channel AQP5 might provide an evidence of reduced salivary function. In this study, we found the presence of mislocalised AQP5 in the CA12 E/K-mutated salivary glands *in vivo*. Thus, to examine whether CA12 and AQP5 physically associate in the salivary glands, we designed this study to assess the effects of CA12 and its E/K mutant on the expression and volume regulation of AQP5. Here, we address the definitive mechanism employed by CA12 and its mutant on the volume regulation of AQP5 with respect to the physiological conditions in the exocrine glands.

## Material and methods

### Reagents and plasmids

β-Actin antibody (A3854), 4,4′-diisothiocyano-2,2′-stilbenedisulfonic acid (DIDS; 462268), carbachol (1092009), acetazolamide (A6011), and amiloride hydrochloride hydrate (Amiloride; A7410) were purchased from Sigma (Saint-Louis, MO). AQP5 (ab92320), Na^+^-K^+^-Cl^−^ co-transporter1 (NKCC1, ab59791), SLC26A6 (ab123001), and AE2 (ab42687) antibodies were purchased from Abcam (Cambridge, MA). CA12 antibody was purchased from ProteinTech Inc. (15180–1-AP; Chicago, IL). 2',7'-Bis-(carboxyethyl)-5-(and-6)-carboxyfluorescein (BCECF)-AM was purchased from TEFlabs (0061; Austin, TX). TNF-α (210-TA-005) was purchased from R&D Systems (Minneapolis, MN). Calcein-AM was purchased from Molecular Probes (C1430; Eugene, OR). Pluronic acid (F-127, 20% in dimethyl sulphoxide, P-3000MP), pHrodo Green AM (P35373), and ZO-1 antibody (940–2200) were purchased from Invitrogen (Carlsbad, CA). All other chemicals were purchased from Sigma. GFP-tagged human AQP5 was a kind gift from Kyung Pyo Park (Seoul National University, South Korea), and DNA plasmids for pcDNA3.0-human NKCC1, mKate-human SLC26A6, HA-rabbit AE2, mKate-human CA12 wild type, and mKate-CA12 E143K mutants were generously provided by Dr. Shmuel Muallem (National Institutes of Health, Bethesda)^8^.

### Cell culture

Human embryonic kidney 293 T cells (HEK293T) was maintained in Dulbecco's modified Eagle's medium (Invitrogen, 11995–065) containing 10% fetal bovine serum (FBS; Invitrogen, 16000–044), and 100 U/mL penicillin–streptomycin (Invitrogen, 15140–122) and incubated at 37 °C in a humidified environment containing 5% CO_2_ and 95% air. When the cells reached 80% confluency, the culture medium was aspirated, and the cells were washed with Dulbecco's phosphate-buffered saline (DPBS, Welgene, South Korea, LB001-02), followed by their treatment with trypsin/ethylenediaminetetraacetic acid (EDTA) for 2 min. The dispersed cells were transferred to new culture dishes for western blotting and co-immunoprecipitation (Co-IP) or to culture dishes with glass coverslips for imaging.

### Isolation of mouse submandibular glands (SMG) acinar cells

All experimental protocols for animals and their maintenance and care were undertaken according to the Gachon University Animal Care guidelines. All animal procedures were approved by the Center of Animal Care and Use, Lee Gil Ya Cancer and Diabetes Institute, Gachon University, and the Institutional Animal Care and Use Committee (IACUC) (Permission number: LCDI-2017–0014). Mouse SMG cells were isolated from 22–25 g C57BL/6 mice, and the isolated tissues and cells were suspended in physiological salt solution A (PSA; 140 mM sodium chloride [NaCl], 10 mM glucose, 5 mM potassium chloride [KCl], 1 mM magnesium chloride [MgCl_2_], 1 mM calcium chloride [CaCl_2_], 10 mM HEPES [pH 7.4], 0.02% soybean-trypsin inhibitor, 0.1% sodium pyruvate, and 0.1% bovine serum albumin [BSA]), and stored on ice until use. The isolated tissues were minced with scissors and incubated in collagenase P solution (2.5 mg/10 ml in PSA; Roche) for 6 min at 37 °C with vigorous shaking. The isolated acinar cells were washed and re-suspended in PSA and stored on ice until use.

### Adenovirus-transduced mice

To overexpress the CA *in vivo*, the adenovirus (Ad)-transduced mice were developed as previously described^8^. Briefly, the CA12 and CA12 E143K fragments were generated by PCR and ligated into pAC-IRES-RFP to generate pAC-CA12-IRES-RFP and pAC-CA12 E/K. Adenoviruses were delivered to 8-week-old C57BL/6 mice. The mice were anesthetised with the both SMG and parotid glands cannulated. Saline (50 μL for the SMG glands) containing AdCA12 or AdCA12 E/K (5 × 10^9^ particles per gland) was then administered by retrograde ductal instillation. After 7 days, the SMG were isolated and dissected for immunostaining.

### Measurement of CBE and NKCC activities

Isolated SMG cells were attached onto coverslips and loaded in the chamber containing 6 μM BCECF-AM in the presence of 0.05% pluronic acid (F-127) in HEPES-buffered solution (Table 1) for 15 min at room temperature. After stabilisation of the fluorescence, the cells were perfused with solution A for a minimum of 5 min prior to measuring the pH_i_. The pH_i_ was measured by BCECF fluorescence at dual excitation wavelengths of 495 and 440 nm and an emission wavelength of 530 nm. The chloride/bicarbonate exchange (CBE) activity of AE2 and SLC26A6 was determined using Cl^−^-free HCO_3_^−^-buffered solution (Table 2) containing 126 mM Na^+^. The cells were incubated with CO_2_-saturated HCO_3_^−^-buffered solution (Table 3) for the acidification of the cytosol, and then perfused with Cl^−^-free HCO_3_^−^-buffered solution. CBE activity was calculated from the slope of increase in pH_i_ during the first 30–45 s in Cl^−^-free HCO_3_^−^-buffered solution and expressed as percent fold change relative to that of the CBE activity of the control. NKCC1 activity was estimated from the slope of change in pH_i_ by using 10 mM NH_4_Cl in HEPES-buffered solution. Administration of NH_4_Cl in the extracellular solution induced initial alkalisation by the diffusion of NH_3_, and then, pH_i_ was decreased by NH_4_^+^ influx, as a substitution of K^+^. The pH_i_ recovery rate in the second phase provides NH_4_^+^ influx. The traces were normalised at the time point of NH_4_Cl administration. The slope of second-phase acidification was calculated and represented as a ratio. To mimic intracellular acidification, the isolated SMG cells were treated with Na^+^-free solution (0Na^+^, Table 4) and Amiloride, a sodium channel inhibitor, followed by 10 mM NH_4_Cl stimulation. Images were obtained at 1-s interval by using a CCD camera (Retiga 6000; Q-Imaging, Canada) linked to an inverted microscope (Olympus, Japan), and analysed with a MetaFluor system (Molecular Devices, PA). Each image was normalised by subtracting the background fluorescence from the raw background signals.

**Table 1.** Composition of HEPES-based solution.

Composition	Final concentration
Sodium chloride (NaCl)	140 mM
HEPES	10 mM
Glucose	10 mM
Potassium chloride (KCl)	5 mM
Magnesium chloride (MgCl_2_)	1 mM
Calcium chloride (CaCl_2_)	1 mM
pH 7.4	
300mOsm (310 for SMG)	

**Table 2.** Composition of Cl^−^-free HCO_3_^−^-buffered solution.

Composition	Final concentration
Sodium gluconate	120 mM
HEPES	2.5 mM
Glucose	10 mM
Potassium gluconate	5 mM
Magnesium sulfate (MgSO_4_)	1 mM
Calcium gluconate	0.5 mM
Sodium bicarbonate (NaHCO_3_)	25 mM
pH 7.8	
310 mOsm	

**Table 3.** Composition of HCO_3_^−^-buffered solution.

Composition	Final concentration
Sodium chloride (NaCl)	120 mM
HEPES	2.5 mM
Glucose	10 mM
Potassium chloride (KCl)	5 mM
Magnesium chloride (MgCl_2_)	1 mM
Calcium chloride (CaCl_2_)	1 mM
Sodium bicarbonate (NaHCO_3_)	25 mM
pH 7.8	
300mOsm (310 for SMG)	

**Table 4.** Composition of Na^+^-free solution.

Composition	Final concentration
NMDG-Cl (pH ≥7.4)	125 mM
HEPES	2.5 mM
Glucose	10 mM
Potassium chloride (KCl)	5 mM
Magnesium chloride (MgCl_2_)	1 mM
Calcium chloride (CaCl_2_)	1 mM
adjusting pH to 7.8 (with. Choline Bicarbonate)	
310 mOsm	

### Cell acidification

To mimic intracellular acidification, the isolated SMG cells were treated with 10 mM NH_4_Cl in the extracellular solution induced initial alkalisation for 1 min and subsequently treated with 0Na^+^ and Amiloride, a sodium channel inhibitor, for indicated times. Treated SMG cells were used in the biotinylated assay, pH imaging, and confocal imaging.

### DNA transfection

Plasmid DNAs (Total amount of DNA: 2 μg) were incubated in 200 μL of Opti-Eagle’s minimum essential medium (Opti-MEM^TM^; Invitrogen, 31985–070) and mixed with the Lipofectamine 2000 mixture. The mixture was incubated at room temperature for 25 min and transferred into cell dishes containing culture medium. After 4 h, the transfected medium was replaced with the fresh culture medium, and the cells were used at 24 h after transfection. Plasmid DNA transfection by Lipofectamine 2000 was performed according to the manufacturer’s protocol (Invitrogen, 11668019).

### Measurement of volume changes

The transfected cells and isolated SMG cells were loaded with 2 μM calcein-AM (Molecular Probes) in the presence of 0.01% pluronic acid (F-127) for 15 min at room temperature. After stabilisation of fluorescence, the cells were perfused with HEPES-buffered solution for a minimum of 5 min prior to volume measurement. The calcein-AM dye was excited at 495 nm, and the emitted fluorescence was measured at 515 nm. Cell volume was determined using a hypotonic (215 mOsm, Table 5) or hypertonic (500 mOsm, Table 6) solution. Fluorescence images were captured at 1-s interval by using a CCD camera (Retiga 6000, Q-Imaging, Canada) linked to an inverted microscope (Olympus, Japan), and analysed with a MetaFluor system (Molecular Devices, PA). Each image was normalised by subtracting the background fluorescence from the raw background signals.

**Table 5.** Composition of hypotonic-buffered solution.

Composition	Concentration
Sodium chloride (NaCl)	80 mM
HEPES	10 mM
Glucose	10 mM
Potassium chloride (KCl)	5 mM
Magnesium chloride (MgCl_2_)	1 mM
Calcium chloride (CaCl_2_)	1 mM
pH 7.4	
215 mOsm	

**Table 6.** Composition of hypertonic-buffered solution.

Composition	Concentration
Sodium chloride (NaCl)	140 mM
HEPES	10 mM
Glucose	10 mM
Potassium chloride (KCl)	5 mM
Magnesium chloride (MgCl_2_)	1 mM
Calcium chloride (CaCl_2_)	1 mM
pH 7.4	
500 mOsm	

### Co-immunoprecipitation, surface biotinylation and Western blotting

Transfected cells were incubated with lysis buffer (Cell Signaling, 9803) containing 20 mM Tris, 150 mM NaCl, 2 mM EDTA, 1% Triton X-100, and a protease inhibitor mixture for 5 min at room temperature. The cells were sonicated and centrifuged at 11,000 ×*g* for 15 min at 4 °C and protein concentration was determined by Bradford assay (Bio-Rad, Hercules, CA). For Co-IP, the lysate was treated with 1 μg/mL of the indicated antibodies at 4 °C for 16 h with gentle shaking and added protein G plus agarose beads (Santa Cruz, SC-2002) for 4 h. The beads were centrifuged at 11,000 ×*g* for 2 min at 4 °C and discard the supernatant and washed thrice with the lysis buffer at 4 °C. The beads were incubated in the sample buffer at 37 °C for 15 min for protein detachment. Eluted proteins were applied in western blotting. To demonstrate the surface expression of proteins, the transfected cells were incubated with 0.5 mg/mL EZ-LINK Sulfo-NHS-LC-biotin (Thermo, 21335) for 30 min on ice, and then treated with 100 mM cold glycine solution (in PBS) for 10 min. The incubated cells were washed thrice with PBS buffer and incubated with the lysis buffer. Cell extracts were centrifuged at 11,000 ×*g* for 15 min at 4 °C. The supernatants were incubated overnight with Avidin beads (Thermo, 20347) at 4 °C and the beads were washed with the lysis buffer. Collected beads were heated at 37 °C for 15 min in the presence of the sample buffer to recover proteins. The warmed protein samples were subjected to separation by using sodium dodecyl sulfate-polyacrylamide gel electrophoresis (SDS-PAGE), and then transferred onto polyvinylidene difluoride (PVDF; Bio-Rad) membranes soaked in methanol. The membrane was blocked with 5% nonfat milk solution in TBS-T (Tris-buffered saline [TBS] and 0.5% Tween-20) for 1 h. The membrane was incubated overnight with the indicated antibodies at 4 °C and washed thrice with TBS-T. Following washing, the membranes were incubated with horseradish peroxidase (HRP)-conjugated anti-mouse and anti-rabbit secondary antibodies, and the protein bands were visualised using the enhanced luminescent solution (Thermo, 32209). Input and β-actin blots were used as loading control.

### Confocal imaging

Transfected cell or sliced SMG tissues were transferred onto cover glasses and fixed with chilled (−20 °C) methanol or 4% paraformaldehyde. The isolated SMG cells were pre-treated with 20 mM NH_4_Cl and 100 μM DIDS in 0 Na^+^ solution for intracellular acidification 1 h before fixation. Fixed cells were treated with 5% goat serum for 1 h at room temperature to block the nonspecific sites. The cells were incubated overnight with primary antibodies (1:50, dilution factor) at 4 °C, followed by washing thrice with PBS. To detect bound antibodies, the cells were treated with goat immunoglobulin G (IgG)-tagged with rhodamine (1:100; Jackson ImmunoResearch, anti-mouse: 115–025-072, anti-rabbit: 111–025-144) or fluorescein isothiocyanate (FITC, 1:100; Jackson ImmunoResearch, anti-mouse: 115–095-071, anti-rabbit: 111–095-003) for 1 h at room temperature. Following incubation, the cells were washed thrice with PBS, and the cover glasses were mounted on glass slides by using Fluoromount-G^TM^ with 4′,6-diamidino-2-phenylindole (DAPI) (Electron Microscopy Sciences, Hatfield, PA, 17984–24). The slides were analysed using a LSM 700 Zeiss confocal microscope (Carl Zeiss, Germany) with ZEN software (Carl Zeiss).

### Statistical analyses

Data from indicated number of experiments were expressed as the mean ± standard error of the mean (SEM). The statistical differences between the mean values obtained from the two sample groups were analysed using Student’s *t*-test parametric. Statistical significance was determined by the analysis of variance in each experiment (*p < .05).

## Results

### Basolateral CA12 mutation disrupts the localisation of AQP5 in salivary glands

**Figure 1.**
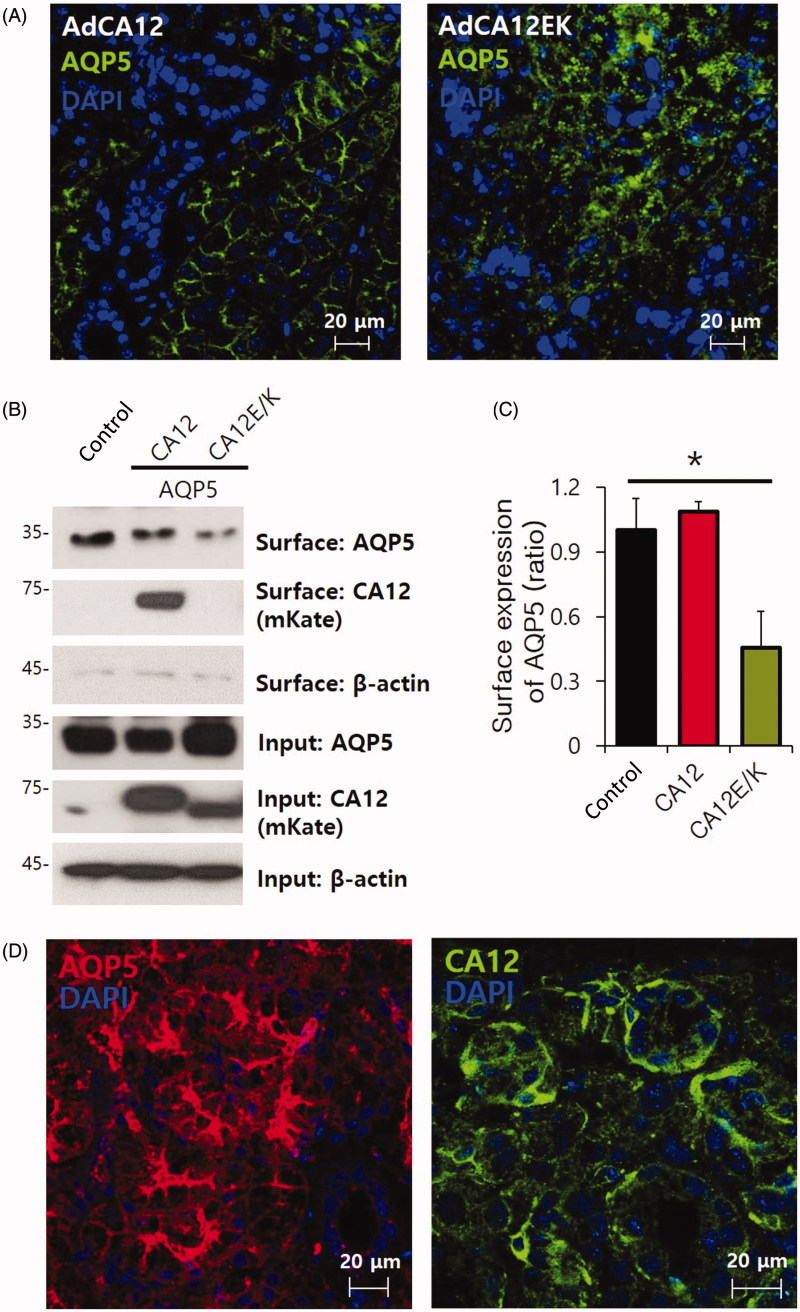
Basolateral CA12 mutation disrupts the localisation of AQP5. (A) Localisation of AQP5 (green) in the submandibular gland (SMG) after delivering the adenoviral CA12 or CA12 E/K. (B) Effect of mKate-CA12 and mKate-CA12 (E/K) on the surface expression of AQP5 in transfected HEK293T cells. (C) The columns represent the mean ± standard error of mean (SEM) of AQP5 surface expression (*p* < .05, indicated by an asterisk, *n* = 4). (D) Native expression of AQP5 (red) and CA12 (green) in mouse SMG.

CA12 is expressed in the basolateral membrane of the acinar and duct cells of the parotid glands^8^. CA12 E143K (E/K) mutation caused reduced secretion of ductal fluids in sealed ducts and inhibited salivation in mice^8^. The total volume of secreted saliva in adenovirus-transduced CA12 E/K was dramatically reduced. In this study, we explored the effect of CA12 and CA12 E/K on the regulation of the water channel AQP5, localised in the luminal membrane as a major water channel in the salivary glands^11–13^ (Supplementary Figure 1). Adenovirus injection was administered to the two SMG and the two parotid glands to enhance the efficacy of viral transfer; the SMG was isolated after 7 days and isolated acinar cells were used for immunostaining. We found mis-localised AQP5 in CA12 E/K-overexpressed salivary glands (Figure 1(A)). To confirm the mis-localisation of AQP5 mediated by CA12 E/K, mKate-CA12 WT and -mutant were over-expressed in HEK293 cells with GFP-tagged AQP5. The CA12 E/K mutation mediated localisation of AQP5 in the cytosol, not in the plasma membrane (Supplementary Figure 2). Surface biotinylated assay also revealed about 60% reduced expression of AQP5 in the presence of CA12 E/K (Figure 1(B,C)). Native AQP5 and CA12 were localised in the luminal and basolateral membrane of the SMG, respectively (Figure 1(D)). Although no physical interaction was observed between CA12 and AQP5 by immunoprecipitation assay, it is not known how basolateral CA12 regulates the expression of luminal AQP5.

### Intracellular pH modulates AQP5 expression in SMG

**Figure 2.**
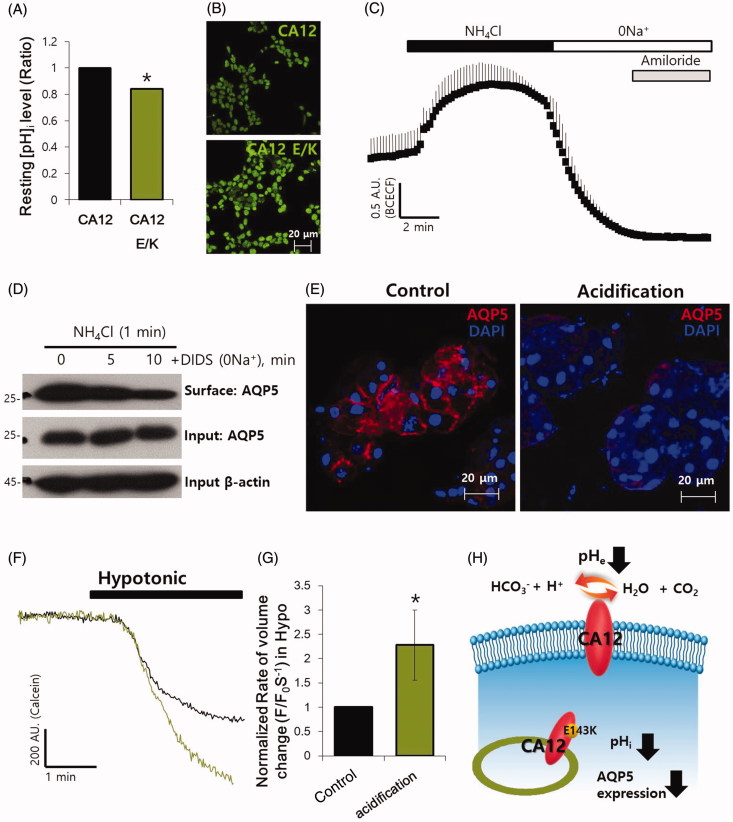
Intracellular pH modulates AQP5 expression in SMG. (A) Analysis of the intracellular resting pH level (ratio) of HEK293T cells transfected with CA12 or CA12 E/K (*p*<.05, indicated by an asterisk, *n* = 7). (B) pHrodo staining (green) images of HEK293T cells transfected with CA12 or CA12 E/K. Enhanced intensity represented acidic pH. (C) pH changes during the NH_4_^+^-pulse acidification technique. Upon removal of 10 mM NH_4_Cl solution after the influx of NH_4_^+^, the cells were acidified using the remaining H^+^. Amiloride (500 μM) was used to inhibit Na^+^ reabsorption through the Na^+^ channel. (D) Surface expression of AQP5 during intracellular acidification with 10 mM NH_4_Cl and 100 μM DIDS in 0 Na^+^ solution (Na^+^-free solution). (E) Changes in AQP5 localisation during intracellular acidification with 10 mM NH_4_Cl and 100 μM DIDS in 0 Na^+^ solution for the indicated time period. SMG acinar cells were stained with DAPI (blue) to indicate the presence of AQP5 (red). (F) Cells were stimulated in a hypotonic (215 mOsm) solution, and the change in volume was measured using 2 μM calcein-AM. The changes in the volume of acidification-induced SMG acinar cells (using 10 mM NH_4_Cl and 100 μM DIDS in 0 Na^+^ solution for 30 min) were measured. The isolated mouse SMG acinar cells were stimulated in the hypotonic (215 mOsm) solution. (G) The columns show the mean ± SEM of the normalised rate of volume change (F/F_0_·S^−1^, *p*<.05, indicated by an asterisk, *n* = 5). (H) Schematic illustration of function and trafficking of CA12 wild type and E/K mutant.

Our previous study showed that CA12 WT is membrane-associated, whereas CA12 E/K mutant can be observed in the cytosol^8^. CAs generate an acidic component, CO_2_^9^, and cytosolic CA12 E/K can be modulated by the intracellular pH (pH_i_). We speculated that fluctuations in the basal pH_i_ level might cause AQP5 localisation. Measurement of the pH_i_ by BCECF showed that the basal pH of CA12 E/K was about 20% lower than that of CA12 WT in HEK293T cells (Figure 2(A)). HEK293T cells were also stained with pH indicator pHrodo-green. Overexpressed CA12 E/K revealed the enhanced intensity, which represented that CA12 E/K-transfected cells were more acidic (Figure 2(B)). We evaluated the effect of pH_i_ on the localisation of AQP5. When the isolated SMG cells were treated with NH_4_Cl, the initial pH_i_ was alkalised by the diffusion of NH_3_ and subsequently acidified by NH_4_^+^ influx. In the presence of amiloride, an inhibitor of amiloride-sensitive NHE and NBC^14^, the pH_i_ moved to the acidic end, and the recovery response was delayed, indicating the inhibition of H^+^ extrusion and HCO_3_^−^ uptake in order to maintain the acidic pH_i_ (Figure 2(C)). We also evaluated the surface expression of AQP5 by using the biotinylated assay at acidified pH_i_. The isolated SMG cells were stimulated in DIDS, followed by NH_4_Cl stimulation. The surface expression of AQP5 was diminished by acidosis (Figure 2(D)). Additionally, the isolated SMG cells were administered NH_4_Cl, and subsequently exposed to DIDS in Na^+^-free solution to acidify the pH_i_, and AQP5 expression was studied using confocal microscopy. Luminal AQP5 expression of SMG acinar cells was almost absent under acidic conditions (Figure 2(E)). These results showed that pH_i_ modulates the membrane expression of AQP5 in SMG. Stimulation of the salivary gland cells results in bulk water efflux and can be observed as cell shrinkage^15^, whereas water influx results in cell swelling. AQP5 has been known as an osmosensor^16^. To evaluate the volume regulation of AQP5 in salivary glands, isolated SMG acinar cells were stimulated in a hypotonic (215 mOsm) solution, and the volume change was measured using the calcein-AM fluorescent technique. Shrinkage of SMG cells and increase in fluorescence intensity were observed when moved to a hypertonic solution, while swelling of SMG cells and decrease in fluorescence intensity were elicited when moved to a hypotonic solution (Supplementary Figure 3A). The mRNA and protein levels of AQP5 at different levels of tonicity did not alter the expression level (Supplementary Figure 3(B,C)). We evaluated the volume regulation of AQP5 under acidified conditions. The isolated SMG cells showed an increase in cell swelling under hypotonic stimulation (Figure 2(F,G)). These results suggest that intracellular acidification reduced the expression of AQP5 and subsequent volume dysregulation. Mutation of CA12 also provided the acidic environment in cytosolic area (Figure 2(H)).

### CA12 E/K mutation modulates the volume regulation of AQP5

**Figure 3.**
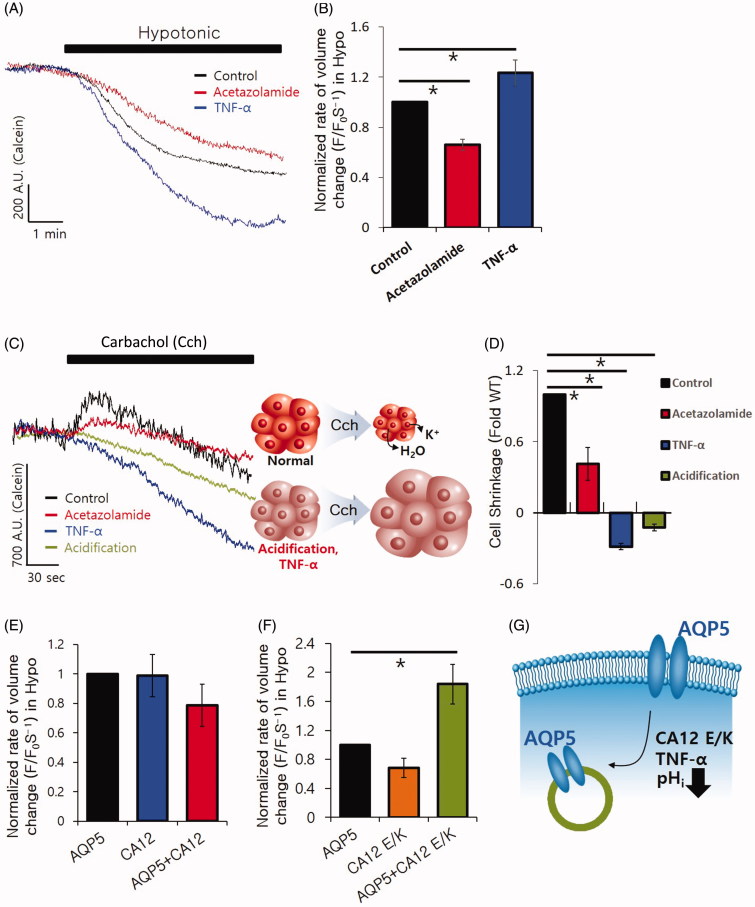
CA12 E/K mutation modulates volume regulation of AQP5. (A) Volume regulation of AQP5 on stimulation with a hypotonic (215 mOsm) solution pre-treated with 100 μM acetazolamide and 10 ng/mL TNF-α for 1 h. (B) The columns represent the mean ± SEM of the normalised rate of volume change (F/F_0_·S^−1^, *p*<.05, indicated by an asterisk, *n* = 4). (C) Volume regulation of SMG on stimulation of 10 μM carbachol with and without treatment with 100 μM acetazolamide and 10 ng/mL TNF-α for 1 h, followed by acidification. (D) The columns represent the mean ± SEM of the fold changes of cell shrinkage after carbachol treatment (*p*<.05, indicated by an asterisk, *n* = 3). (E and F) Volume regulation of AQP5 by stimulation in a hypotonic (215 mOsm) solution with and without CA12 and CA12 E/K. The columns represent the mean ± SEM of normalised rate of volume change (F/F_0_·S^−1^, *p*<.05, indicated by asterisk, *n* = 6). (G) Schematic illustration of cross talk mechanism to affect AQP5 localisation.

In response to inflammation, the transport of salt and water is decreased to provide physiological protection^17^^,^^18^. The stimulation of TNF-α, a primary component of inflammation, reduces saliva secretion. Isolated SMG cells were pre-treated with TNF-α to confirm the volume regulation occurring during inflammation. We found that TNF-α mediated the increase in cell swelling through the attenuation of AQP5 (Figure 3(A,B)). We administered the CA inhibitor acetazolamide, a well-known diuretic^19^^,^^20^. Acetazolamide inhibited the swelling property of cells (Figure 3(A,B)). To confirm the effect of acidification and acetazolamide stimulation on the swelling property of cells, isolated SMG cells were stimulated using the cholinergic agonist carbamyl chlorine chloride (carbachol) to activate fluid secretion (Figure 3(C)). Stimulation of fluid secretion by carbachol results in large water efflux and cell shrinkage in the salivary glands^15^. Isolated SMG cells were found to shrink by carbachol stimulation. However, in the presence of intracellular acidification and TNF-α stimulation, isolated SMG cells did not shrink and the volume was maintained (Figure 3(D)), suggesting that the loss of AQP5 caused volume dysregulation in the presence of the agonist of fluid secretion.

To determine whether CA12 E/K mutation mediates volume dysregulation, we performed volume measurement in CA12 and AQP5-overexpressed HEK293T cells. The volume of CA12 WT-enriched cells was not modulated by the hypotonic stimulation (Figure 3(E)). The hypotonic solution elicited an increase in the cell swelling property in the presence of CA12 E/K mutation, suggesting dysfunction of the cytosolic AQP5 induced by CA12 E/K mutation in response to hypotonicity (Figure 3(F)). These results suggest that the endogenous acidosis caused by inflammatory signals, including CA12 E/K mutation, mediate concomitant internalised expression of AQP5 and obviously dysregulate the volume in salivary glands.

### AQP5 is functionally associated with the transepithelial transporter AE2

**Figure 4.**
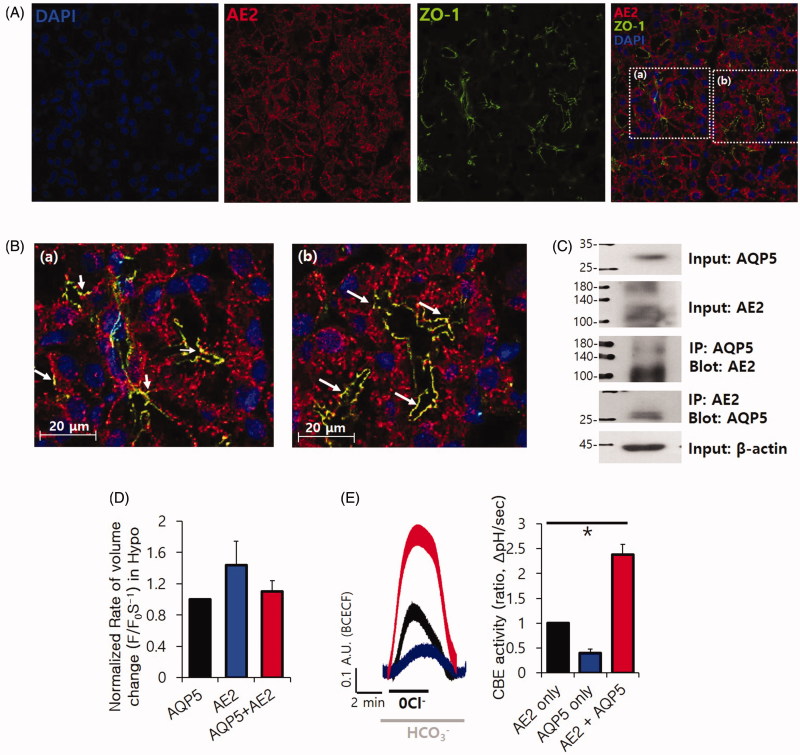
AQP5 is functionally associated with the transepithelial transporter AE2 (A) Native localisation of DAPI (blue), AE2 (red), and ZO-1 (green) in the mouse SMG. (B) High-magnification images of the co-staining areas in (a) and (b). (C) Co-immunoprecipitation (Co-IP) of AE2 and AQP5 in isolated SMG. (D) Volume regulation of AQP5 with and without AE2 by hypotonic stimulation in HEK293T cells. The columns represent the mean ± SEM of the normalised rate of volume change (F/F_0_·S^−^^1^, *n* = 5). (E) Effect of AQP5 on the chloride/bicarbonate exchanger (CBE) activity of AE2-transfected HEK293T cells. The columns represent the mean ± SEM of CBE activity (*p* < .05, indicated by an asterisk, *n* = 3).

To the best of our knowledge, AQP-mediated fluid movement is regulated by an osmotic gradient^21^. To provide a basis for understanding the role of AQP5 in transepithelial ion transport, we examined whether these solute transporters modulate volume regulation of AQP5 in the salivary glands. Various transporters are expressed and involved in salivary function. Among them, AE2 was found to be modulated by CA12, in our previous study^8^. The AE2 localised in the plasma membrane and several dots of AE2 co-localised with the tight junction marker ZO-1 (Figure 4(A,B)). The direct interaction of membrane proteins indicate that these proteins are functionally linked^16^. We confirmed the protein–protein interaction of AE2 and AQP5 in SMG cells and an overexpressed system in HEK293T cells (Figure 4(C), Supplementary Figure 4A). The volume regulation of AQP5 was independent of AE2, whereas AE2-overexpressed cells showed enhanced swelling property (Figure 4(D)). We next evaluated the changes of pH_i_ by using the BCECF-AM to measure the HCO_3_^−^-transporting activity. Interestingly, AE2 activity was enhanced in the presence of AQP5 (Figure 4(E)).

### AQP5 is functionally associated with the transepithelial transporter NKCC1

**Figure 5.**
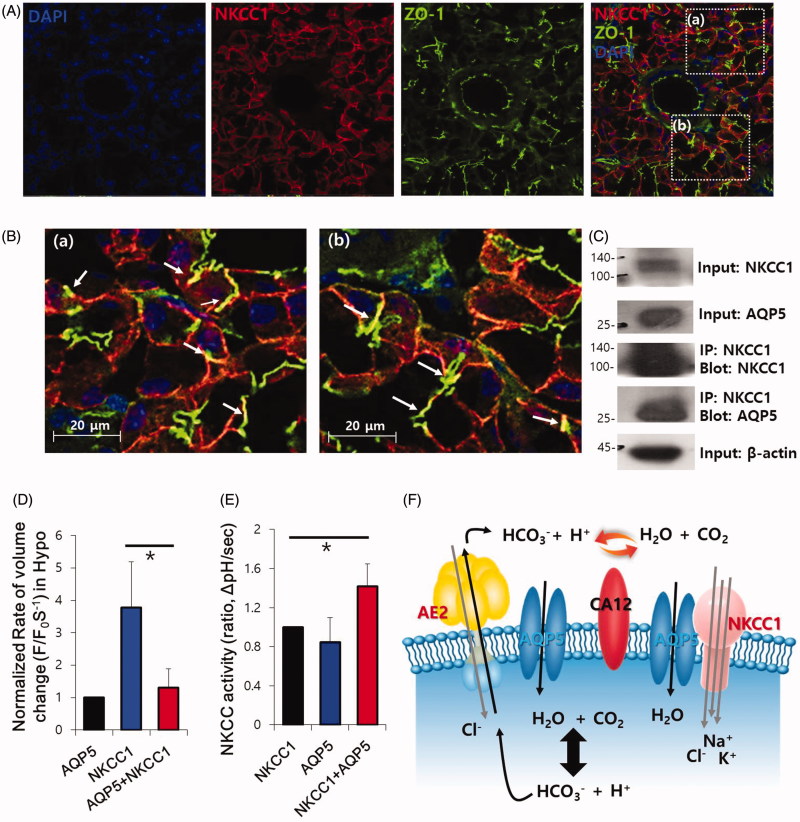
AQP5 is functionally associated with the transepithelial transporter NKCC1. (A) Native localisation of DAPI (blue), NKCC1 (red), and ZO-1 (green) in the mouse SMG. (B) High-magnification images of the co-staining areas in (a) and (b). (C) Co-IP of NKCC1 and AQP5 in SMG. (D) Volume regulation of AQP5 with and without NKCC1 by hypotonic stimulation in HEK293T cells. The columns represent the mean ± SEM of the normalised rate of volume change (F/F_0_·S^−1^, *p*<.05, indicated by an asterisk, *n* = 5). (E) Effect of AQP5 on the NKCC1 activity of NKCC1-transfected HEK293T cells. The columns represent the mean ± SEM of NKCC activity (*p*<.05, indicated by an asterisk, *n* = 3). (F) Schematic illustration of cross talk mechanism between AQP5 and the transepithelial transporters AE2 and NKCC1.

To assess the relationship between transepithelial ion transport and volume regulation, we confirmed that another solute-transporting system NKCC1 is physiologically associated with AQP5. NKCC1 and AQP5 co-localised in the lumen of acinar cells and interacted with each other (Figure 5(A–C)). We confirmed the protein–protein interaction of NKCC1 and AQP5 in an overexpressed system of HEK293T cells (Supplementary Figure 4B). Volume regulation of AQP5 was maintained with and without NKCC1 (Figure 5(D)). AQP5 also enhanced NKCC activity, like AE2 activity (Figure 5(E)). It is noted that the solute transporters-overexpressed system, including AE2 and NKCC1, which only exists in native water channels, enhanced the swelling property (Figures 4(D) and 5(D)). Collectively, these results suggest that the activities of AE2 and NKCC were supported by AQP5 for osmotic regulation, suggesting that the membrane expression of AQP5 affects the swelling property in the presence of solute influx. Normal expression of CA12 also provides HCO_3_^−^ consumption and water source to transporters and AQP5, respectively (Figure 5(F)). The Cl^−^-HCO_3_^−^ exchanger AE2 and solute carrier transporter family 26 (SLC26) A6 are abundantly expressed in the plasma and luminal membranes of salivary glands, respectively, as acid loaders^22^. Another luminal protein SLC26A6 was independent of AQP5, with no changes in activity or protein–protein interaction (Supplementary Figure 5).

## Discussion

Dysregulated volume homoeostasis in the salivary glands plays an important role in the secretion of fluid and saliva. AQP5 is involved in transcellular water permeability and is a dominant water channel in the epithelia of the salivary and lacrimal glands, and the lung alveoli^1^^,^^2^. Appropriate expression of AQP5 in the luminal membrane aids homoeostatic volume regulation. In this study, we found that the regulatory factors, endogenous acidification, inflammatory factors, and CA12 E/K mutation, all mediate the dysregulation of AQP5. Basically, decreased AQP5 expression might reduce water intake. However, enhanced cell swelling by decreased AQP5 expression in the plasma membrane might indicate ion transporter-mediated volume dysregulation in the absence of AQP5. Movement of solutes by ion transporters is accompanied by the movement of water through AQP5. Intrinsic modulation by acidosis might affect the expression of AQP5 in the plasma membrane.

The present findings show that the effect of the CA12 E/K mutation, expressed in the cytosol, mediated the mistargeting of AQP5. The membrane-associated CAs promote extracellular acidification^23^. The mislocalised CA12 E/K in the cytosol can mediate aberrant pH_i_ homoeostasis. Our results provide strong evidence that AQP5 expression is sensitive to pH_i_. Abnormal localisation of CA12 E/K mutation generates an acidic intracellular environment, and pH_i_-sensitive components such as AQP5 can be affected. We speculated that reduced expression of AQP5 by CA12 E/K provided an ion transporter-rich state in the plasma membrane. The movement of solutes by ion transporters such as AE2 and NKCC1 can be acutely diluted by water to prevent osmotic imbalance. The cross-talk between ion transporters and AQP5 might maintain complementary volume regulation. The osmotic maintenance of AQP5 with transepithelial ion transporters is a typical process in volume regulation. Disruption of AQP5 expression by CA12 E/K can provide another candidate mechanism for reduced salivary volume.

Human CA12 mutation, which results in autosomal recessive hereditary hyperchlorhidrosis, reduces CA activity and leads to an autosomal-recessive phenotype, including excessive Cl^−^ secretion in sweat^24^. CAs are involved in the reverse reaction involving CO_2_ and water to release H^+^ and HCO_3_^−^^9^. Mammalian CAs are identified in the cytoplasmic (CA2 and CA7), mitochondrion-associated (CA5), secretory (CA6), and plasma membrane-associated (CA4, CA9, CA12, and CA14) enzymes^9^. The plasma membrane-localised CAs functionally couple with and regulate the activity of HCO_3_^−^ transporters, including NBCe1 and AE2^8^^,^^25^^,^^26^. Although the functional role of CAs in fluid and HCO_3_^−^ secretion, including AQP5, is obvious, the functional and physiological interactions between other type of CAs and AQP5 are still unclear.

In this study, we, for the first time, to the best of our knowledge, provided direct evidence of the modulatory role of CA12 on AQP5 expression. Dysregulation of AQP5 by the CA12 deficiency caused by CA12 E/K mutation might involve excessive Cl^−^ secretion, resulting in hyperchlorhidrosis in humans. In addition, for the cancer treatment, several blockades of oncogene CA12 have been developed^27–29^. Accordingly, intracellular acidification, inflammation, or application of blockades of oncogene CA12 should be induced osmotic dysregulation in case of volume-sensitive exocrine glands such as the salivary glands, which abundantly express AQP5.

## Supplementary Material

AQP5-V20_vertical_r3_supple.pdf

## References

[1] Matsuzaki T, Suzuki T, Koyama H, et al. Aquaporin-5 (aqp5), a water channel protein, in the rat salivary and lacrimal glands: Immunolocalization and effect of secretory stimulation. Cell Tissue Res 1999;295:513–21. 10022971 10.1007/s004410051257

[2] Hoffert JD, Leitch V, Agre P, King LS Hypertonic induction of aquaporin-5 expression through an erk-dependent pathway. J Biol Chem 2000;275:9070–7. 10722758 10.1074/jbc.275.12.9070

[3] Ma T, Song Y, Gillespie A, et al. Defective secretion of saliva in transgenic mice lacking aquaporin-5 water channels. J Biol Chem 1999;274:20071–4. 10400615 10.1074/jbc.274.29.20071

[4] Delporte C, Bryla A, Perret J Aquaporins in salivary glands: from basic research to clinical applications. Int J Mol Sci 2016;17:166. 10.3390/ijms17020166 PMC4783900 26828482

[5] Jensen HH, Login FH, Park JY, et al. Immunohistochemical evalulation of activated ras and rac1 as potential downstream effectors of aquaporin-5 in breast cancer in vivo. Biochem Biophys Res Commun 2017;493:1210–6. 28958942 10.1016/j.bbrc.2017.09.125

[6] He Z, Dong W, Hu J, Ren X Aqp5 promotes hepatocellular carcinoma metastasis via nf-kappab-regulated epithelial-mesenchymal transition. Biochem Biophys Res Commun 2017;490:343–8. 28619511 10.1016/j.bbrc.2017.06.046

[7] Ren Y, Lu H, Reinach PS, et al. Hyperosmolarity-induced aqp5 upregulation promotes inflammation and cell death via jnk1/2 activation in human corneal epithelial cells. Sci Rep 2017;7:4727. 28680052 10.1038/s41598-017-05145-y PMC5498491

[8] Hong JH, Muhammad E, Zheng C, et al. Essential role of carbonic anhydrase xii in secretory gland fluid and HCO_3_ (-) secretion revealed by disease causing human mutation. J Physiol 2015;593:5299–312. 26486891 10.1113/JP271378 PMC4704518

[9] McKenna R, Frost SC Overview of the carbonic anhydrase family. Sub-Cell Biochem 2014;75:3–5. 10.1007/978-94-007-7359-2_1 24146371

[10] Gresz V, Kwon TH, Hurley PT, et al. Identification and localization of aquaporin water channels in human salivary glands. Am J Physiol Gastrointest Liver Physiol 2001;281:G247–54. 11408278 10.1152/ajpgi.2001.281.1.G247

[11] Gresz V, Kwon TH, Gong H, et al. Immunolocalization of aqp-5 in rat parotid and submandibular salivary glands after stimulation or inhibition of secretion in vivo. Am J Physiol Gastrointest Liver Physiol 2004;287:G151–61. 14988067 10.1152/ajpgi.00480.2003

[12] Verkman AS, Matthay MA, Song Y Aquaporin water channels and lung physiology. Am J Physiol Lung Cell Mol Physiol 2000;278:L867–79. 10781416 10.1152/ajplung.2000.278.5.L867

[13] Kreda SM, Gynn MC, Fenstermacher DA, et al. Expression and localization of epithelial aquaporins in the adult human lung. Am J Respir Cell Mol Biol 2001;24:224–34. 11245621 10.1165/ajrcmb.24.3.4367

[14] Li J, Koo NY, Cho IH, et al. Expression of the Na+-HCO_3_^−^ cotransporter and its role in phi regulation in guinea pig salivary glands. Am J Physiol Gastrointest Liver Physiol 2006;291:G1031–40. 16782694 10.1152/ajpgi.00483.2005

[15] Teos LY, Zhang Y, Cotrim AP, et al. IP3R deficit underlies loss of salivary fluid secretion in Sjogren's syndrome. Sci Rep 2015;5:13953. 26365984 10.1038/srep13953 PMC4568516

[16] Procino G, Milano S, Tamma G, et al. Co-regulated pendrin and aquaporin 5 expression and trafficking in type-b intercalated cells under potassium depletion. Cell Physiol Biochem 2013;32:184–99. 24429825 10.1159/000356638

[17] Weidenfeld S, Kuebler WM Cytokine-regulation of Na(+)-K(+)-Cl(-) cotransporter 1 and cystic fibrosis transmembrane conductance regulator-potential role in pulmonary inflammation and edema formation. Front Immunol 2017;8:393. 28439270 10.3389/fimmu.2017.00393 PMC5383711

[18] Hosoi K Physiological role of aquaporin 5 in salivary glands. Pflugers Arch 2016;468:519–39. 26537593 10.1007/s00424-015-1749-6

[19] Nguyen HV, Stuart-Tilley A, Alper SL, Melvin JE Cl(−)/HCO_(3)_^(−)^ exchange is acetazolamide sensitive and activated by a muscarinic receptor-induced [Ca(2+)](i) increase in salivary acinar cells. Am J Physiol. Gastrointestinal Liver Physiol 2004;286:G312–20. 10.1152/ajpgi.00158.2003 12958022

[20] Imiela T, Budaj A Acetazolamide as add-on diuretic therapy in exacerbations of chronic heart failure: A pilot study. Clin Drug Investig 2017;37:1175–81. 10.1007/s40261-017-0577-1 PMC5684277 28965280

[21] Zhang J, Yan M, Gu W, et al. Downregulation of aquaporins (aqp1 and aqp5) and Na,K-ATPase in porcine reproductive and respiratory syndrome virus-infected pig lungs. Inflammation 2018;41:1104–14. 29532265 10.1007/s10753-018-0762-2

[22] Boron WF, Chen L, Parker MD Modular structure of sodium-coupled bicarbonate transporters. J Exp Biol 2009;212:1697–706. 19448079 10.1242/jeb.028563 PMC2683013

[23] Bouchareb R, Cote N, Marie Chloe B, et al. Carbonic anhydrase xii in valve interstitial cells promotes the regression of calcific aortic valve stenosis. J Mol Cell Cardiol 2015;82:104–15. 25771146 10.1016/j.yjmcc.2015.03.002

[24] Feldshtein M, Elkrinawi S, Yerushalmi B, et al. Hyperchlorhidrosis caused by homozygous mutation in CA12, encoding carbonic anhydrase XII. Am J Hum Genet 2010;87:713–20. 21035102 10.1016/j.ajhg.2010.10.008 PMC2978943

[25] Becker HM, Klier M, Deitmer JW Carbonic anhydrases and their interplay with acid/base-coupled membrane transporters. Sub-Cell Biochem 2014;75:105–34. 10.1007/978-94-007-7359-2_7 24146377

[26] Orlowski A, De Giusti VC, Morgan PE, et al. Binding of carbonic anhydrase IX to extracellular loop 4 of the NBCe1 Na+/HCO_3_^−^ cotransporter enhances NBCe1-mediated HCO_3_^−^ influx in the rat heart. Am J Physiol Cell Physiol 2012;303:C69–80. 22538240 10.1152/ajpcell.00431.2011

[27] Guler O, Simone G, Supuran C Drug design studies of the novel antitumor targets carbonic anhydrase ix and xii. Curr Med Chem 2010;17:1516–26. 20166929 10.2174/092986710790979999

[28] Singh S, Lomelino CL, Mboge MY, et al. Cancer drug development of carbonic anhydrase inhibitors beyond the active site. Molecules 2018;23:1045 (22 pages). 10.3390/molecules23051045 PMC6099549 29710858

[29] Waheed A, Sly WS Carbonic anhydrase xii functions in health and disease. Gene 2017;623:33–40. 28433659 10.1016/j.gene.2017.04.027 PMC5851007

