# Supplementary material for: Carbonic anhydrase 12 mutation modulates membrane stability and volume regulation of aquaporin 5
Source: J Enzyme Inhib Med Chem. 2018 Nov 19;34(1):179–88. doi: 10.1080/14756366.2018.1540475 (PMC6249555; doi:10.1080/14756366.2018.1540475)
Supplement: AQP5-V20_vertical_r3_supple.pdf [file IENZ_A_1540475_SM8091.pdf]

Carbonic anhydrase 12 mutation modulates membrane stability and volume regulation of aquaporin 5

Soyoung Hwang[1*], Jung Yun Kang[2*], Min Jae Kim[2], Dong Min Shin[2]†, and Jeong Hee Hong[1]†

[1]Department of Physiology, College of Medicine, Department of Health Sciences and Technology, GAIHST, Gachon University, 155 Getbeolro, Yeonsu-gu, Incheon 21999, Republic of Korea; [2]Department of Oral Biology, BK21 PLUS Project, Yonsei University College of Dentistry, Seoul 03722, Republic of Korea

Running title: Effect of CA 12 mutation on AQP5

Key words: Carbonic anhydrase 12, aquaporin 5, volume regulation, acidosis, salivary glands

* Authors have equally contributed to this article.

† Correspondence to DMS (dmshin@yuhs.ac) and JHH (minicleo@gachon.ac.kr)

Authors:

Soyoung Hwang

[1]Department of Physiology, College of Medicine, Gachon University, 155 Getbeolro, Yeonsu-gu, Incheon 21999, Republic of Korea

Email: snrntlwy1004@naver.com

Jung Yun Kang

[2]Department of Oral Biology, BK21 PLUS Project, Yonsei University College of Dentistry, Seoul 03722, Republic of Korea

Email: hannahkang77@gmail.com

Min Jae Kim

[2]Department of Oral Biology, BK21 PLUS Project, Yonsei University College of Dentistry, Seoul 03722, Republic of Korea

Email: mjkim0789@naver.com

†Dong Min Shin, DDS, PhD

[2]Department of Oral Biology, BK21 PLUS Project, Yonsei University College of Dentistry, Seoul 03722, Republic of Korea

Phone: +82-2-2228-3051; Fax: +82-2-364-1085; E-mail: dmshin@yuhs.ac

†Jeong Hee Hong, PhD

[1]Department of Physiology, College of Medicine, Gachon University, 155 Getbeolro, Yeonsu-gu, Incheon 21999, Republic of Korea

Phone: +82-32-899-6682; Fax: +82-32-899-6039; E-mail: minicleo@gachon.ac.kr

Supplementary Figure 1. Hwang and Kang et al.

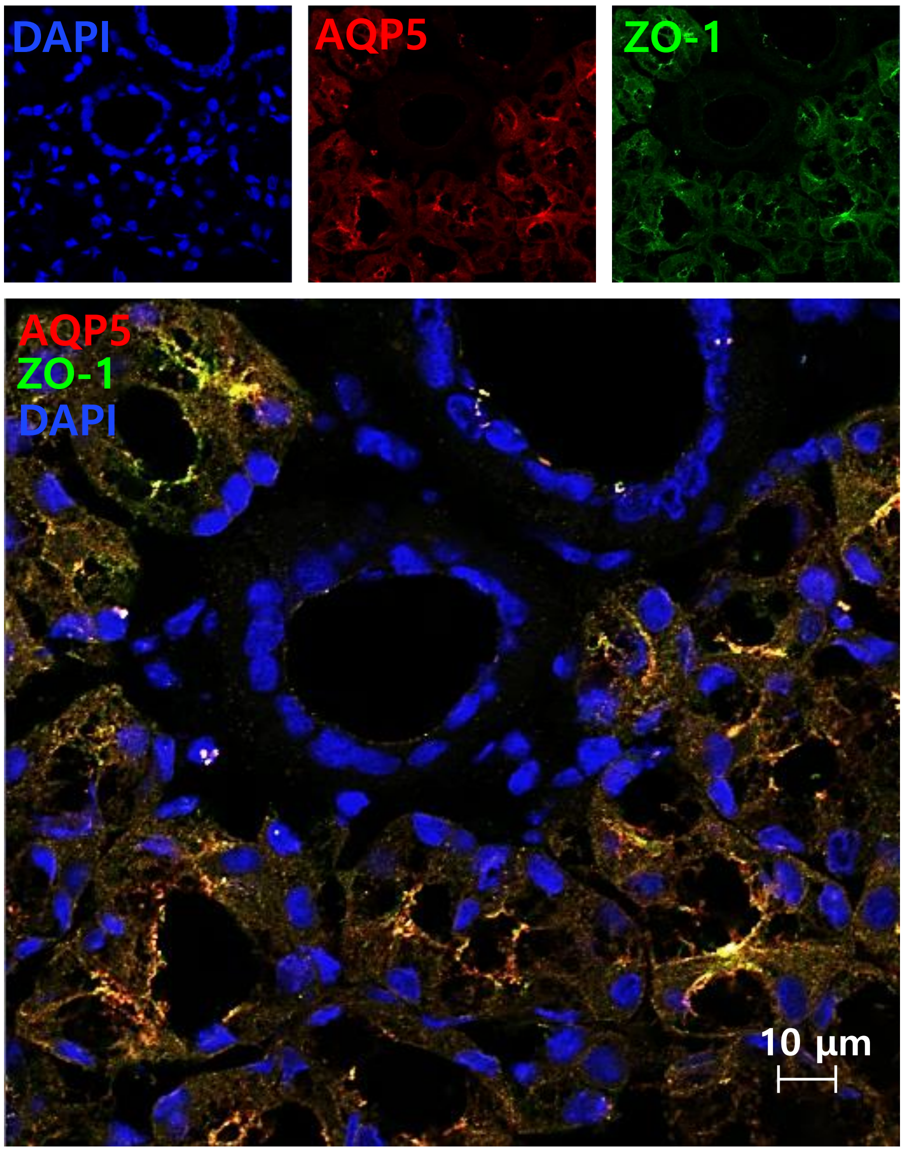

Supplementary Figure 1. Native localization of DAPI (blue), AQP5 (red), and ZO-1 (green) in the mouse SMG

Supplementary Figure 2. Hwang and Kang et al.

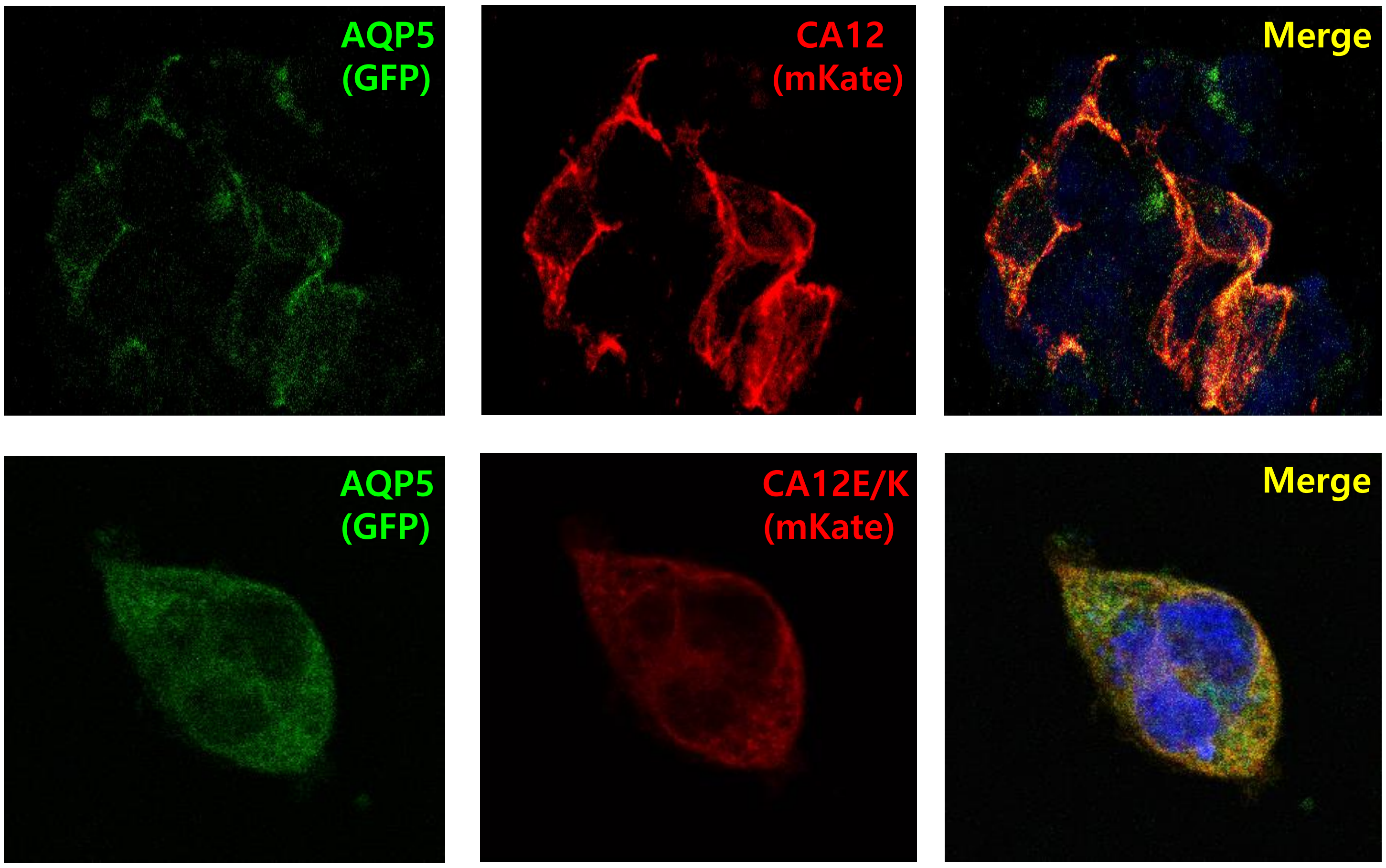

Supplementary Figure 2. mKate-CA12 (upper, red) and mKate-CA12 E/K (lower, red) co-localised with the GFP-tagged AQP5 in transfected HEK293T cells

Supplementary Figure 3. Hwang and Kang et al.

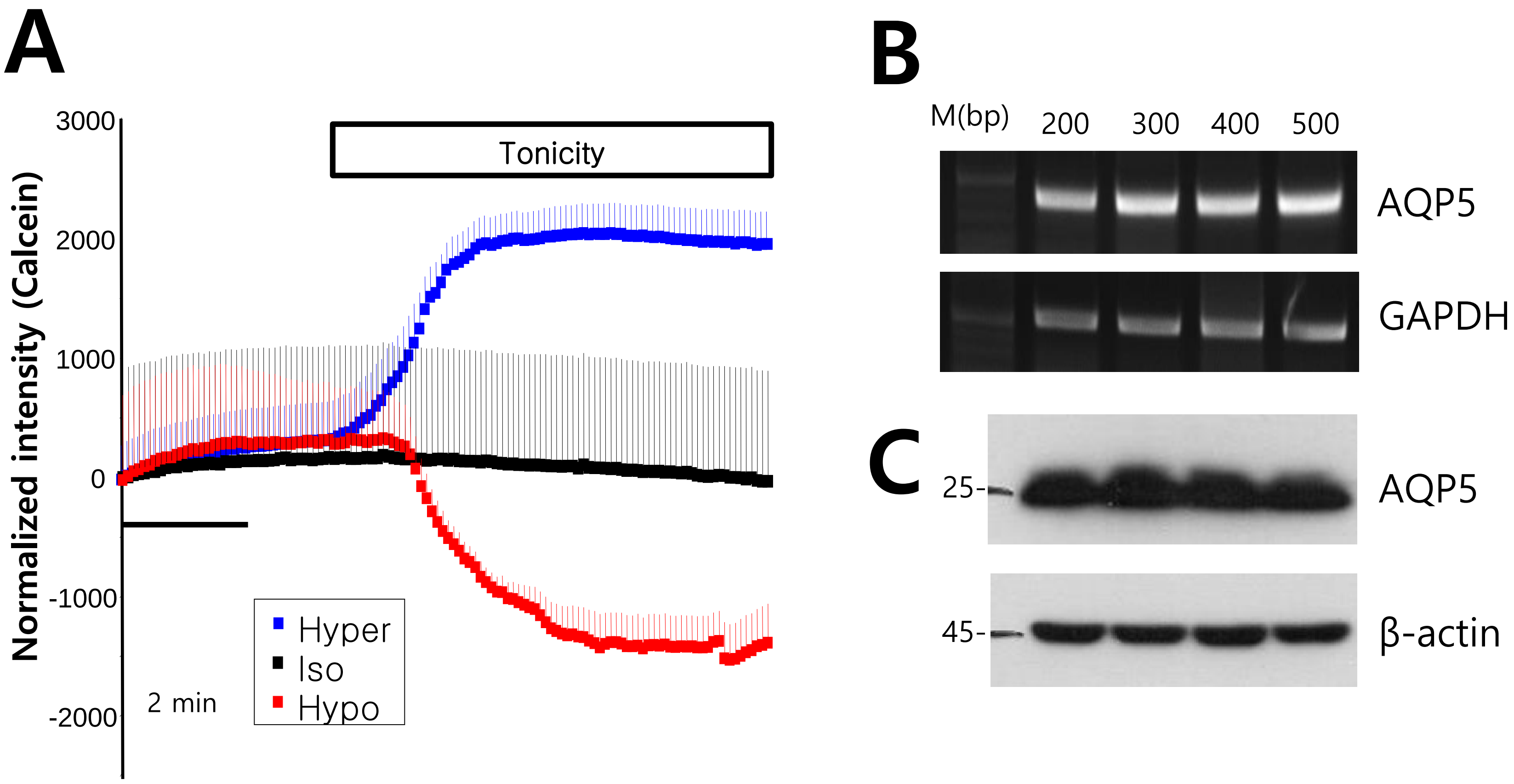

Supplementary Figure 3. (A) Cell volume changes of mouse SMG acinar cells as induced by hyper (500 mOsm)/iso (310 mOsm)/hypotonicity (215 mOsm) stimulation. (B) Analysis of PCR products of AQP5 and (C) protein expression level of AQP5 stimulated with different levels of osmolarity for 30 min

Supplementary Figure 4. Hwang and Kang et al.

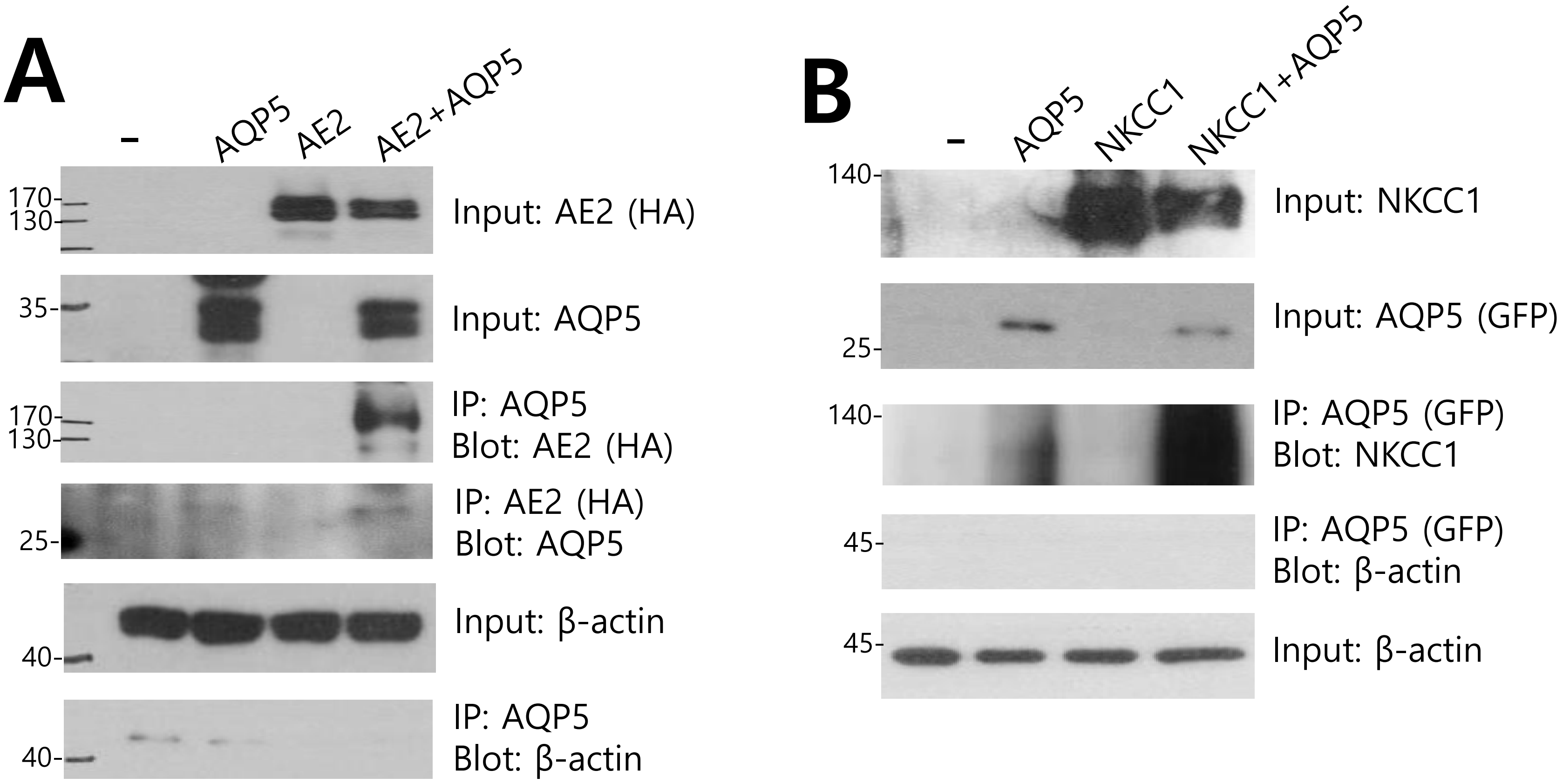

Supplementary Figure 4. (A) Co-IP of AE2 with AQP5-expressed HEK293T cells. The cells were transfected with AQP5 and HA-tagged AE2 and immunoprecipitated with AQP5 antibody and blotted with HA antibody. Reversed Co-IP of AE2 and AQP5 was performed with HA antibody and blotted with AQP5 antibody. (B) Co-IP of NKCC1 with AQP5-expressed HEK293T cells. Cells were transfected with GFP-tagged AQP5 and NKCC1 and immunoprecipitated with GFP antibody and blotted with NKCC1 antibody. Input blots and β–actin blots were used as the loading controls.

Supplementary Figure 5. Hwang and Kang et al.

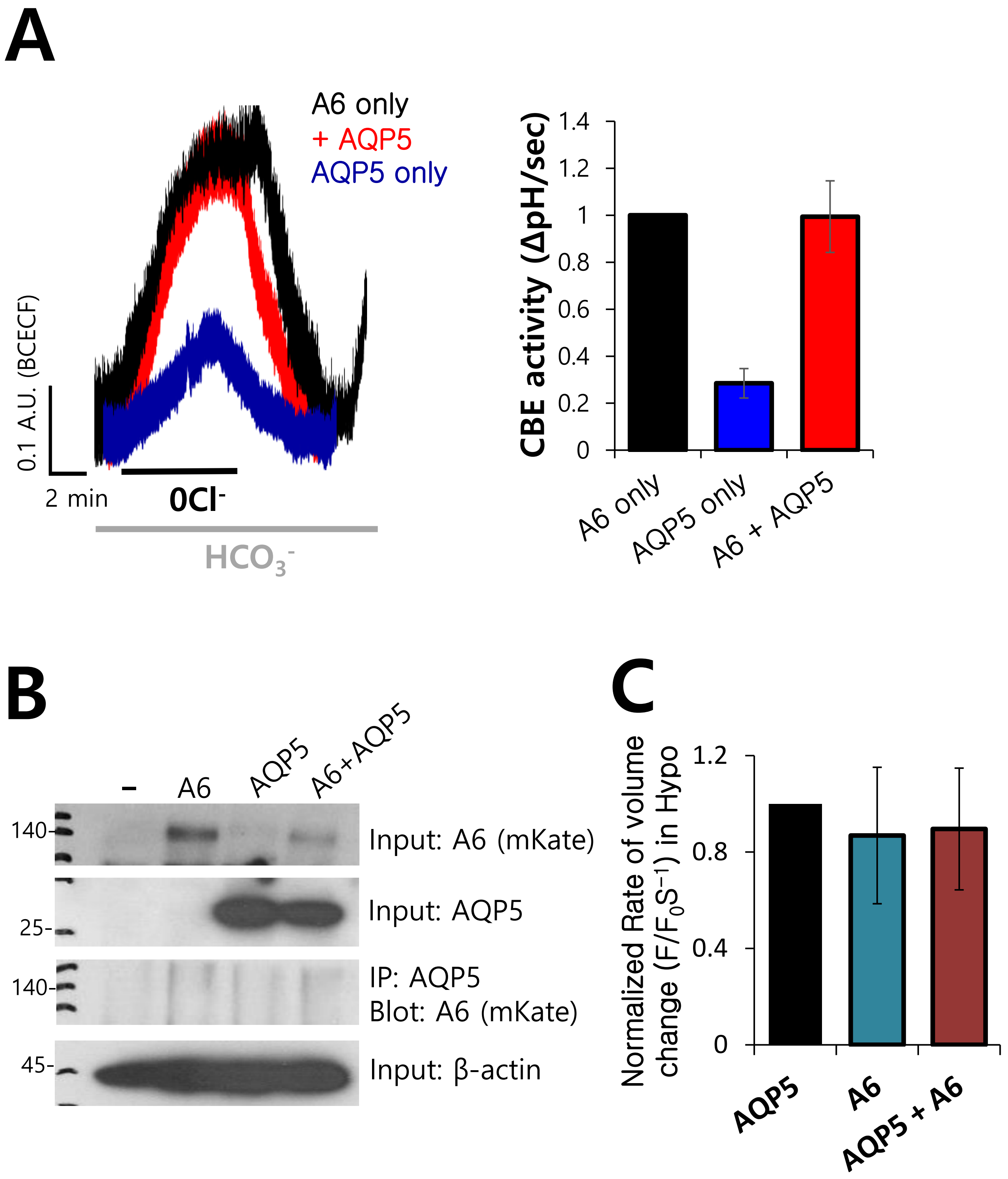

Supplementary Figure 5. (A) Effect of AQP5 on the chloride/bicarbonate exchanger (CBE) activity of mKate-SLC26A6-transfected HEK293T cells. The columns represent the mean ± SEM of CBE activity. (B) Co-IP of SLC26A6 and AQP5 in HEK293T cells. Cells were immunoprecipitated with AQP5 antibody and blotted with mKate antibody. (C) Volume regulation of AQP5 with and without SLC26A6 by hypotonic stimulation in HEK293T cells. The columns represent the mean ± SEM of the normalized rate of volume change ($F/F_0{\cdot}S^{-1}$).
